# The Endoscope, and Its Application to the Diagnosis and Treatment of Urinary Affections

**Published:** 1867-06

**Authors:** A. J. Desormeaux


(*Continued from page 208.*)
with reference to their symptoms. In the case of each, there is much pain, and considerable discharge of the same appearance in the acute stage; in the chronic stage, the pains are absent, the secretion scarcely observable, unless some accidental cause occurs to increase the affection. Thus the disease may often pass unperceived until its grave effects are made evident.

The affections most frequently complicating blennorrhagia are orchitis or epididymitis. Since my attention has been called to this subject, and I have been enabled to closely investigate the characteristics of blennorrhœa, I have not seen a single case of epididymitis connected with urethral discharge, unless such discharge was connected with bulbous, membranous, or prostatic granulous inflammation. Under the effect of catheterisms, the testicle often becomes painful and inflamed, but this inflammation is generally slight, and when it does become severe is generally caused by irritation due to the passage of sounds. Epididymitis, does not come on until the blennorrhagia takes up its chronic character. You have studied it in this phase, and it is needless for me to speak to you more about it, but let us look for a moment to the course of granular urethritis in its full chronic state. It may present its acute or chronic symptoms so far as granulations are concerned. The first subject of urethral granulation which fell under my observation, complained only of epididymitis, the blennorrhœal cause was so ancient that it dated back eleven years.

In such cases, where the specific symptoms are not readily perceived, it not rarely occurs that orchitis is attributed to mechanical irritation or to epidemic influence, most frequently, however, the epididymitis, of blennorrhagic origin, is as chronic as its cause. It may continue for an indefinite time, apparently relieved, again to appear, taking on a sub-acute character and simulating tubercule of the testicle. Often have I seen such an error committed, and that too by distinguished surgeons, and without the aid of the endoscope it may occur to all and at any time.

A person affected with clap, so slight as not to be perceived, may be taken, without apparent cause, with scrotal pain, the epididymis becomes swollen, effusions often form in the vaginal tunic, and the skin becomes red; but instead of increasing, all of these symptoms soon decrease; however, pain of slight degree continues for some days, and the tumefaction may remain for two, three, or even more weeks. At length the inflammation disappears, but the testicle long remains tender, and the swelling leaves behind an induration, not so well determined as in inflammatory epididymitis. We may conceive that the cure is perfected, but it is only temporary; it may continue for a greater or less length of time, be it weeks or years, but suddenly the disease reappears, follows the same phases, and, in again disappearing, leaves a fresh inodulation, the seat of which is not so well defined as in the acute affection. The result is, that after several relapses, the testicle takes on a condition peculiar and not easily described (*englobi dans une masse bossiler*), or in other words, it becomes more or less a tuberculous or nodular mass, bound together, hard, often insensible, then painful, and then increasing in size.

When the disease commences, it is more or less similar to a case of tubercular orchitis; later on, it is difficult to tell if or not it is a case of tuberculous testicle. The only circumstance that can be used as a diagnostic remedy for this error, is the fact that tubercles rarely last so long without becoming softened and ulcerated. But it is not rare that these symptoms are so simple, they sometimes become more serious, the inflammation may become phlegmonous, in them there is formed a swelling of red appearance, shortly becoming fluctuant, which opens spontaneously or is opened by art, pus escapes, the inflammation subsides, the pus becomes serous, and the opening, for weeks, remains fistulous. I have seen many such cases. Years ago, such cases were pronounced tuberculous, and only after long study have I been able to correct an error into which I had fallen. It must be conceded that the symptoms in these cases are more or less deceptive. Let me call your attention, however, to the nature of the pus, as bearing upon correct diagnosis; cicatrization is slower than in softened tubercles; and, lastly, after cure of blennorrhagic orchitis we do not find these hard cords which enable us to trace tubercular fistulas, even long after they are cured. This, however, does not furnish sufficient ground upon which to base a diagnosis. The watery discharge from the urethra, be it ever so little, might be relied upon, but, unfortunately, when tubercle takes hold of the testicle, it often, at the same time, attacks the prostate, and this causes a discharge similar to that in simple granular urethritis.

Now, the endoscope comes into play and clears up the question, brings light out of darkness; there can be no doubt as to presumptive signs, when we are able to see the granular erosions in the urethra. In such cases, with a sure diagnosis, the treatment is readily fixed upon—combat the testicular engorgement, and attack the disease in the canal. In every case that I have seen, the epididymitis ceased upon the cure of the urethral granulations.

We will now resume the history of blennorrhœa. The endoscope furnishes us the means of recognizing the characteristic local lesion, and by its aid we are enabled to separate it from affections simulating it in their exterior symptoms, not very defined. Once the disease well defined and disembarrassed from all that obscures its history, we are enabled to decide upon its march, its termination, and its peculiar complications, and also to distinguish between it and such affections as may simulate it. Now we will pass on to the study of its etiology.

The different authors assign many causes for blennorrhœa and blennorrhagia, about the same thing. First, comes contagion, or intercourse with an affected person, excessive intercourse with a well person, onanism, traumatic irritation, catheism, irritating injections, any and all female discharges, chancrous pus, irritating or relaxing food, and especially asparagus, which I think most innocent, drinks, such as beer and tea, and some such medicines as cantharides. You may add to these the internal causes, such as the development of the teeth, worms, dartrous affections, certain different temperaments, the different seasons, and variations of temperature.

We might say that the causes of blennorrhagia are as multiple as general pathology reports in its chapter on etiology, and we may well be astonished that every one is not affected with it, or, at least, that it is not more frequent than coryza, which recognizes but one cause. If we properly appreciate these causes, and apply to them the knowledge we possess, we will perceive that some have only an imaginary influence, and others, though capable of producing a urethral discharge, do not give rise to specific blennorrhagia, either acute or chronic. This can only be brought about by contagion, by direct contact with the fluid secreted by a similar nature.

I have shown you how I became acquainted with urethral granulations, the characteristic anatomical lesion of blennorrhagia or its synonym, granular urethritis. I have been able, often, to recognize the presence of granulations in the urethra and in the uterine neck, in cases of persons cohabiting together, and reason has taught me that in such cases they were contagious, even when I was first acquainted with the work of M. Thiry, of which I have spoken. This learned surgeon, who had in view, especially, diseases of the eye, recognized the contagious character of conjunctival granulations. M. Wecker, and other ophthalmologists, admit this contagion, but as one of the numerous causes of trachoma, whilst M. Thiry considers it as the only cause, and he has seen it carried not only from one eye to another, but also transferred from the genitals to the eyes. Accidents have shown to him that this disease of the conjunctiva is produced by the inoculation of the muco-pus furnished by the urethra, or uterine granulations. By way of experiment, he has transposed the muco-pus from the eye to the urethra and also to the uterine neck, and the result has been the appearance, in each case, of similar erosions. I do not think he was supplied with means to examine the interior of the canal, and, consequently, could only infer the existence of a disease identical in its nature, producing granulations in different organs susceptible of attack, and proved that these granulations were the result of a contagion whose agent was the granular virus (*virus granuleux*).

M. Thiry's ideas and mine are alike, only his ideas are based upon a more general and direct experience. Mine are founded upon clinical observation, aided by the endoscope.

My first observations demonstrated to me granulations caused (*remontant*) by blennorrhagia. I also found this affection the basis of all granular urethritis, and when I followed it by the aid of the endoscope, as you can also do, I saw the development of the granulations at a certain period of the disease. Often, at the Lourcine Hospital, in patients affected with blennorrhagia, granulations of the uterine neck have visibly formed. You may have often observed this, and I have already called your attention to the fact that non-specific affections of the neck do not give rise to it. Hence, the natural indication is, that blennorrhagia, blennorrhœa, and granular urethritis are the same disease. This disease is transmitted by a virus, a virus which is the active hypothetical principle of the contagious pus; it might be called granular virus, but as we generally distinguish the virus by the produced disease, and not by the anatomical lesions, I prefer the term blennorrhagic virus (*virus blennorrhagic*).

In its acute stage, the contagion is by no means doubtful; but such is not the case in the chronic state. Unfortunately, blennorrhagia is not generally regarded as contagious, and hence it results that the evil, wherever its seat may be, in the conjunctiva, the urethra, or the uterine neck, propagates itself, unless the necessary precautions are used. This contagion is certain, and especially in acute blennorrhagia. M. Thiry has demonstrated that, be the secretion of conjunctival granulations as feeble as possible, it is inoculable as soon as enough of it can be collected; and, with my experience, I can state that the chronic form of the granular affection is transmissible from one sex to the other. From man to woman, I have so often seen uterine granulations appear, as a consequence of granular urethritis, that no doubt can exist, in my mind, on the subject; from the woman to the man, many times, I have seen patients affected with blennorrhagic discharge, contracted from women who were not exposed, and who were only subject to a slight leucorrhœa, and in whom I have been able to find, by aid of the speculum, evident marks of granular metritis. So the granular affection, in its chronic state, is inoculable, and though such is frequently the fact, it would occur much more often if the secretion was not so much attenuated as sometimes to be incapable of reproduction. You can readily imagine that after the canal is well washed by the flow of urine, it may require several hours for the discharge to become so virulent as to be able to reproduce the disease, in other words, to become inoculable.

The chronic affection often transmits its chronic virus; but it sometimes gives rise to acute blennorrhagia, most frequently, however, from various causes, or from the excitation accompanying the act of infection, the disease takes on its acute character and thus transmits it to healthy persons. The return of the affection from chronic to acute is not a necessary result, for we often see blennorrhœa producing blennorrhagia. I have seen several examples of it, but will only mention the following, because the circumstances caused me to doubt the possibility of transmission:—

T., aged twenty-eight, an employé, after several badly treated blennorrhagias, was subject to a slight continuous gleet, accompanied by a tickling sensation in the urethra. These symptoms always increased after connection. Having gone through several different courses of treatment, he took, for two days, Chopart's lotion, and then entered the hospital with an orchitis. Of the testicular affection he was soon cured, and left with the blennorrhœa still on him. Some months after, persuaded that the discharge, scarcely visible, could not be contagious, he married. Shortly after, I was called to see the bride, a woman of irreproachable character, and I at once saw she had one of the most violent cases of blennorrhagia I had ever seen. The vulva, the vagina, and the uterine neck were all attacked, and in every point accessible to sight, there were the superficial erosions characteristic of lost epithelium, the loss caused by blennorrhagic inflammation. The diagnosis was very evident. After a few days, granulations commenced to appear on the uterine neck. When I saw the husband, he was only affected with a blennorrhœa, for which he had previously consulted me. He assured me his disease had never been followed by acute sequences, which, moreover, could not have disappeared in that time; by an endoscopic examination, I found extensive granulations in the bulbous portion of the urethra. Thus, we see that a blennorrhœa, without retaining its acute character, has caused a true blennorrhagia, or, if you prefer the expression, a chronic granular urethritis, the contagion of which produced upon the woman the acute granular affection.

If we take a retrospect of what we have said upon the etiology of blennorrhœa, we must conclude that it is always the result of contagion, whether it commences in its acute or chronic form, or if the virus is blennorrhœic or blennorrhagic. If other causes than contagion have been assigned, it is that different affections have been confounded, but the endoscope always shows the characteristic granulations. We have already seen that, in these cases, the discharge is often kept up by an herpetic or arthritic cause. But, perhaps, the error is more frequently caused by the fact that the patient was the subject of granulations which might be called latent, and which excitement might bring into the acute stage; and thus may be naturally explained those cases quoted of true blennorrhagia contracted by intercourse with a healthy person. In addition, I have often seen persons believing themselves cured of an old affection, who dated their present attack to only a few days back, but the endoscope has shown, in such cases, granulations of a date evidently anterior.

There is, however, a block here, gentlemen; some of you may tell me that you have observed granular metritis in cases where blennorrhagic influence could not be suspected. How do you know this? Your patients were above suspicion. Are you very sure that their husbands had not preserved an old discharge, of slight sweating character, the result of former affection, forgotten and difficult to discover? Have you examined them? Are you sure there were no granulations in the deep-seated portions of the urethra? For my part, I am sure that your patients are affected with granulations, and I am equallv certain they have been contracted by contagion.

But call to mind, if you please, what I told you in my last lecture, and see if you have not mistaken for these granulations others of a fleshy kind, caused by a very natural, but very different species of complaint, the appearance of which may be changed by accident, or may you have been deceived by an optical delusion, such as I have advised you of, and of which I was long the victim, and which makes us mistake the granular concave depressions of an herpetic eruption for hemispheric enlargements. At present, I will drop this subject, as it will necessarily come up again when we take up the uterus; only I will call upon the testimony of my own students to testify that they have rarely seen granular metritis since they have been able to recognize herpetic ulcerations on the uterine neck.

Before stating a treatment for a disease, it is necessary to have it defined, to eliminate all that can simulate it, because, no matter what name it takes on, in these affections of different natures, it is easy to understand that what is well in one case is bad in another; we mean that, generally, therapeutics become more simple as the disease, better studied, disconnects itself from all that might be confounded with it. When I was a student of medicine, one of our professors said that, "the more remedies proposed to cure any one disease, it was sure that none good had been found;" and this just reflection is appropriate to such a disease as the blennorrhœa of the authors, in which they have united many different affections having only one common symptom and one common seat. If you are of the opinion of this professor, you must believe that there is no good remedy against blennorrhœa. I will not mention here all the various articles used in injections, nor the different formulas of cubebs, copaiva, or turpentine, doubtless very good when the disease has lost its violence, but which I have never seen cure, the granulations once established. These authorities have been imposed upon by the fact that the remedies used temporarily checked the discharge, and thus thought it to be cured; but for my part, I have always seen it recur upon the cessation of the treatment. Question your patients, and you will learn that most of them have uselessly employed such means during months, and sometimes years. The inability of ordinary treatment led to irritating injections, called caustic, in hopes of producing a new inflammation to supersede the original chronic one, and thus to bring about a cure. This mode has been pronounced successful, I have never seen it so; and, often, patients tell us that after cruel suffering, when the artificial inflammation ceased, the blennorrhœa reäppeared with increased vigor. It is easy to account for this want of success; in fact, the injections are too weak to modify the granular surface, and too painful for the patient to submit to them as often and as continuously as may be necessary, for it must be borne in mind that granulations yield only after a long time. The inability of these rational means has caused the admission of others, the constant failure of which has alone been able to preserve. It has been stated that a new blennorrhagia in its acute form will overpower an old one, and that both will cure together. It is difficult to understand such an idea; for my part, the result of my observation shows that the patient who grafts a blennorrhagia upon an old blennorrhœa is very fortunate if, the blennorrhagia passed, he does not find himself worse off than before. It has also been stated that, once the disease has reached its entire chronic state, it may be cured by cohabitation; some authors add, that all contagion has disappeared. The discharge is always contagious, as much so as is granular ophthalmia; and, besides, occasion is not wanting to the result of this practice, so pleasing to the patient. Never have I seen a cure by such advice, but, on the contrary, a few days will show the disease to be aggravated and prolonged. Thus we say, by force, that urethral granulations are not subject to ordinary therapeutics as a means of cure. This compels us to agree with Chomel, who said of uterine granulations, "they will not yield except to certain topical remedies, and especially to cauterization." Once knowing the lesion which keeps up blennorrhœa, it should be easy to treat it. It is the same that applies to other granular affections, such as are successful in granular metritis or conjunctivitis, in other words, cauterization.

How should this cauterization be practiced? To this end, strong injections have been employed, even of nitrate of silver passed into the canal in its fused state. We have already spoken of injections; they are too feeble to act efficiently, and too painful to be continued, and, if made more strong, they would increase rather than diminish the disease, by attacking healthy parts. So far as the porte caustic goes, it blindly attacks all parts, those well and those affected, and this the surgeon cannot foresee; even the granulations, if it should reach them, are but slightly affected, too strong at one place and nothing at another. I must add, that I do not believe that any surgeon is sufficiently courageous, or any subject sufficiently patient to submit to such treatment, as long as might be necessary for a cure.

What we cannot find out by ordinary means, the endoscope will afford us surely and easily. The instrument, made to bear upon the diseased parts, permits us to judge of the portion attacked and to cauterize it; it is enough to reach the diseased parts and then to apply the caustic direct, thus preventing its contact with others not affected. I prefer, as a caustic, the nitrate of silver, one which I have often used in like diseases of the womb, after having tried many others. The liquid caustic has the advantage of reaching the anfractuosities, of acting upon all without touching any too much. I have tried the solid stick, applied through the sound of the endoscope, but it burned too deeply, and caused eschars upon the points touched, whilst others were not affected in this way. So it has the inconvenience of acting incompletely, and of causing loss of substance, and of thus causing the patient to become a victim of almost certain stricture. All of these I have abandoned, and now use the azotate of silver in solution, say from one-third to equal portions. (℞. Azot. arg. chrys. 5 or 15 grammes to 15 grammes of water.) This solution never produces eschars, it is rather catharitic than caustic, and, if I do use the word caustic in speaking of its employment, it is only that none other, more appropriate, has yet been adopted.

The application is very simple:—When the lesion to be attacked has been discovered at the end of the sound, clean it well with dry cotton wool, carried in on a probe more or less long, then, in like manner, cotton saturated with the solution may be applied directly to the parts and left in contact with them for some moments before being withdrawn. This operation should be employed whenever granulations are found. The points touched immediately become whiter and the granulations more apparent. One fact, well worthy of mention, is, that the patients experience but slight pain; frequently they only complain of slight heat in the touched part. M. Wecker also makes the same remark in reference to the cauterization of the conjunctiva. After each cauterization the emission of urine is painful, but this pain, always bearable, though sometimes severe, diminishes with each discharge, and most frequently disappears on the following day. Cold lotions, applied externally, tend much to calm it. It is, moreover, necessary to bear in mind that a certain degree of inflammation is necessary for the destruction of the granulations. At the commencement of the treatment, the cauterizations should be repeated every three or four days; later, when the granulations have disappeared and only a roughened surface, caused by erosion, once a week will be often enough to apply them. If the ulceration is covered with fleshy granulations, especially if they are fungous and easily bleed, they should be touched with a strong solution; in such cases, equal parts of nitrate of silver and distilled water become necessary.

As a consequence of this cauterization, I have but rarely met with a slight febrile attack, or a commencing orchitis. A little sulphate of quinine will meet all the exigencies of the first, without interrupting the treatment; for the second, I have never been called upon to suspend the cauterizations for any length of time.

Repeated cauterization is undoubtedly the most essential part of the treatment, without which a cure cannot be obtained, still it is well to join to it other adjuvants, which are not wanting in efficacy and utility. In uterine and conjunctival granulations, injections and collyria are useful aids; so injections into the urethra should be added to the cauterization, though the frequent washing of the canal by the urinal discharge renders it less necessary in the urethra than in the vagina. I have tried many injections and have concluded the almost exclusive adoption of the decoction of roses of Provins (a French town) the best. Though of slight astringency, this injection seems to be capable of producing as much effect as more powerful astringents, and it has, besides, calming qualities which diminish the pain of the treatment. It may be said, in addition, that, if some of it should reach the bladder, no harm will result, and, after what we have said as to the seat of the granulations, it is evident that an injection limited only to the anterior portion of the urethra is simply useless. When the granulations have disappeared and a kind of watery discharge alone continues, stronger astringent injections may be advantageously used. In such cases, I employ sulphate of zinc, acetate of lead, alum, krameria, and tannin, in one word, all the substances employed in like cases, but of a weak strength. Baths, also, are useful. I generally recommend one to be taken after each operation. They bring back the local and general calmness, disturbed by cauterization, and most especially in nervous patients.

Generally, I do not advise any interior treatment. I have tried diuretics and alkalines, for the purpose of acting upon the local affection through but without avail, unless the liquid (urine) is so charged with salts as to be unusually irritating. In such cases, mineral water, of an alkaline character, to my thinking, is the best.

As for diet, such as is generally used is the best, and, if necessary, tonic, in weak or lymphatic cases; liquor, wine, and strong coffee are to be avoided. These excitants are always injurious. I have often seen the case set back by improper indulgence.

But this is not all, sexual intercourse *must* be forbidden, not only a little, but any. A single infraction of this law will often materially interfere with the cure, and loses all the good done by previous treatment. I have often seen that when the patients were not continent, simply by the inspection of an ulceration, previously in the way of rapid cure, thrown back, very red, and bleeding at the slightest touch. I have also seen patients keep up the disease for a long time, and render all treatment inert, until they have consented to be utterly abstinent in all sexual relations. Thus, you will see how I differ from those who recommend this as a curative means.

This treatment, well and thoroughly carried out, all of your cases will be cured. Up to the present, I have yet to see the first resisting case, when it has been sufficiently continuous; but you must bear in mind that the cure requires a long time, so inform your patients and demand their aid. The granular affection does not change its character in changing its seat, and in the urethra, as in the conjunctiva, or in the uterus, a cure must not be expected in less than two or three years. (*Sic.*—Is it not months? TRANS.)

To fix the period for the cessation of the treatment, is a delicate matter; as long as the sound is introduced, or nitrate of silver is applied, so long the discharge will not cease, injections even will keep it up to a certain extent. The only guide is the appearance of the ulceration. When the mucous membrane has retaken its naturally smooth and polished appearance, though still slightly red, when the nitrate only pales without much whitening its surface, all cauterizations may be stopped. Then I continue, for a short period, injections, soon to quit them, for the purpose of keeping up only the hygienic treatment before spoken of, and that as long as possible. The discharge soon ceases of itself.

Up to the present time, we have only studied discharges due to granular affections. I do not, however, wish to stop without a few words on the treatment of herpetic and arthritic affections. I have already shown you how the endoscope exposes true blennorrhagia; you will naturally recollect that, without the aid of this instrument, the distinction between the different discharges is impossible, and you can only reach them by presumption.

The diagnosis in such cases is still more difficult and important, from the fact that the herpetic affection often follows granular urethritis. We often see herpetic plates developed upon the vulva, the vagina, the glans penis, and the prepuce as a consequence of vaginal blennorrhagia and balano-posthitis, or of the irritation kept up in those parts by chancre. The relation existing between these diseases is, one acts as the cause of the consequence, and causes the second; it does not less certainly follow that a discharge continues and distresses the patient, who cannot tell the difference which has taken place in his condition; moreover, the herpetic discharge is one to be rid of. In this discharge, the treatment should not be the same as in granular urethritis. In one, the general treatment is secondary in effect; in the herpetic, it is primary. It is necessary to attack the dartrous element at once, and by the most appropriate remedies; alkaline baths, depuratives, alkaline mineral waters, Fowler's solution, etc. I do not propose to insist upon the same treatment that may be found necessary in herpetic and arthritic affections located differently. As a local application, the nitrate of silver does not do well in these cases; frequently I have seen it increase rather than diminish the disease. So I have quit its use and now employ the oil of juniper, (*cade*,) applied to the herpetic plates, the effect of which seems to be favorable. As for injections, astringents seem to answer well; but the best are composed of oil of cade and oil of almonds, compounded in the proportion of one-fifth or one-tenth of the oil of cade, otherwise, employ mild alkaline injections; sulphurous injections are also beneficial. Heat, completely inert in granular urethritis, is very useful in this form of the disease.

I must press upon your attention here, the fact that blennorrhœa is an important element in many severe affections of the urethra, and also because the endoscope furnishes means of distinguishing granular urethritis from other discharges.

In some patients, of a soft and lymphatic temperament, the mucous membrane of the urethra and of the vagina becomes the seat of hypersecretion, which may often simulate blennorrhagia. The endoscope will aid us to a correct diagnosis. Astringent injections will stop the discharge in such cases, but it will reappear on the cessation of the treatment. The only rational treatment is the use of general and generous tonics, and, when possible, sea-bathing, stimulating mineral waters, reconstituents, sulphurous, iodic, or saline; the choice of these must depend upon the temperament of the patient.

I have treated long upon this disease, blennorrhœa, because of its important bearing upon many urethral diseases, and because the endoscope will enable us to study it by a new light, showing us the granulations giving rise to the discharge, and enabling us to apply directly the proper remedy. We must now study the strictures it produces, when not arrested sufficiently early. In my next lecture, I will take up that subject and demonstrate to you the use of the endoscope in the premises, as to exploring and treating confirmed strictures.

Up to the present time we have been entirely occupied with the consideration of granular urethritis, and no mention has been made of strictures as a consequence. I have kept back their history, so as to present it all at once, and draw the line of demarcation between those and others of a traumatic character, lesions, different in their origin but like in their consequences, symptoms, and indications of treatment.

If, in investigating blennorrhagia, and the consequent strictures, as a single affection, commencing at the time of contagion, you will necessarily be led to the conclusion that there are three different grades in the march of the disease, each of which tend to stricture.

The first stage is blennorrhagia, or *acute inflammatory stricture.* The last makes a fibrous, inodular, organic stricture.

Intermediate, between these, a condition occurs, which prepares the transition from the first to the third. As Alph. Robert said, in his report to the Medical Academy, in 1852:—"*Between the acute primitive inflammation, characterized by redness and swelling of the membrane of the urethra, and the consecutive stricture, characterized by a fibrous cicatrix, there is a period transitory, which is neither inflammatory or that of properly so-called stricture.*"

This period, indicated by Alph. Robert, can only be recognized by sight; to do so, the endoscope is essentially necessary. It has taught us the existence of a granular urethritis, which could only be taught by an instrument like the endoscope. Let us rapidly run over the three different species of stricture, one following the other, and all preceding the inodular.

During the acute period of blennorrhagia, the swelling of the mucous and subjacent textures form a decided temporary stricture, and the flow of the liquid is so painful that it causes a spasm, adding to the difficulties of emission. Thence, a complete retention occurs. It may happen that the passage of the urine becomes impossible, and the interruption become complete; we call this *acute inflammatory stricture.* Except in such cases, rare, which demand a decided and especial treatment, catheterism was all that was necessary.

It so happens, however, without going back to granular urethritis that blennorrhagia gives rise to permanent stricture. In these cases, the inflammation in some points becomes intense, and is readily observed externally by its painful nodosities. The tissues are too profoundly changed to again take on their natural formation; sometimes the urethra becomes inextensible and forms what is often called a chordee, the cord breaks, and the urethra is torn. In each case, strictures form, which become inodular; these strictures occur early; they are more or less spasmodic in character; sometimes persistent and then, again, retractile; they are organized under the influence of much irritation, and the inodular tissue retracts in the irritated parts as in a burn. At this period, so far as the endoscope shows us, the urethritis has not reached the bulb, but is confined to the spongy portion of the urethra. This, I think, will fully explain the remarks of many surgeons, who have stated that stricture of the spongy part of the urethra was more difficult to dilate, and more liable to be torn than in other parts of the canal. In other respects, all strictures are of an inodular character.

Granular urethritis once set up, we have seen how it continues the swelling of the urethral walls, and the inflammatory retraction of their fibrous elements; the resulting stricture may well be called *chronic inflammatory stricture.*

